# The effect of spinal versus general anaesthesia on perioperative muscle weakness in patients having bilateral total hip arthroplasty: a single center randomized clinical trial

**DOI:** 10.1186/s40001-023-01435-6

**Published:** 2023-10-20

**Authors:** Sam Van Boxstael, Laurens Peene, Dimitri Dylst, Joris Penders, Admir Hadzic, Ingrid Meex, Kristoff Corten, Dieter Mesotten, Steven Thiessen

**Affiliations:** 1 Critical Care Department, Ziekenhuis Oost-Limburg, Schiepse Bos 6, 3600 Genk, Belgium; 2 Faculty of Medicine and Life Sciences & Limburg Clinical Research Center, UHasselt, Diepenbeek, Belgium; 3 Laboratory of Clinical Biology, Ziekenhuis Oost-Limburg, Genk, Belgium; 4 Department of Orthopaedic Surgery, Ziekenhuis Oost-Limburg, Genk, Belgium

**Keywords:** Perioperative muscle weakness, Spinal anaesthesia, General anaesthesia, Bilateral total hip arthroplasty, Neuro-endocrine stress response

## Abstract

**Background:**

Perioperative neuro-endocrine stress response may contribute to acquired muscle weakness. Regional anaesthesia has been reported to improve the outcome of patients having total hip arthroplasty. In this study, it was hypothesized that spinal anaesthesia (SA) decreases the perioperative neuro-endocrine stress response and perioperatively acquired muscle weakness (PAMW), as compared to general anaesthesia (GA).

**Methods:**

Fifty subjects undergoing bilateral total hip arthroplasty (THA) were randomly allocated to receive a standardized SA (*n* = 25) or GA (*n* = 25). Handgrip strength was assessed preoperatively, on the first postoperative day (primary endpoint) and on day 7 and 28. Respiratory muscle strength was measured by maximal inspiratory pressure (MIP). Stress response was assessed by measuring levels of Adrenocorticotropic hormone (ACTH), cortisol and interleukin-6 (IL-6).

**Results:**

Handgrip strength postoperatively (day 1) decreased by 5.4 ± 15.9% in the SA group, versus 15.2 ± 11.7% in the GA group (*p* = 0.02). The handgrip strength returned to baseline at day 7 and did not differ between groups at day 28. MIP increased postoperatively in patients randomized to SA by 11.7 ± 48.3%, whereas it decreased in GA by 12.2 ± 19.9% (*p* = 0.04). On day 7, MIP increased in both groups, but more in the SA (49.0 ± 47.8%) than in the GA group (14.2 ± 32.1%) (*p* = 0.006). Postoperatively, the levels of ACTH, cortisol and IL-6 increased in the GA, but not in the SA group (*p* < 0.004).

**Conclusion:**

In patients having bilateral THA, SA preserved the postoperative respiratory and peripheral muscle strength and attenuated the neuro-endocrine and inflammatory responses.

*Trial registration*: clinicaltrials.gov NCT03600454.

**Supplementary Information:**

The online version contains supplementary material available at 10.1186/s40001-023-01435-6.

## Background

PAMW may cause short-term functional impairment [1]. Pathophysiological mechanisms and preventive strategies are not known, although PAMW has some similarities with intensive care unit acquired weakness (ICUAW). Increased release of catabolic hormones and cytokines (neuro-endocrine stress response) are among the proposed mediators in the complex pathophysiological process of ICUAW and could be responsible also for PAMW [2, 3]. THA elicits the greatest stress response next to cardiac surgery and major abdominal and vascular surgery [4]. The surgically induced neuro-endocrine stress response can be detrimental and leads to systemic inflammatory response syndrome (SIRS), hyper-metabolism, and hyper-catabolism associated with postoperative complications such as muscle wasting, impaired immune function and wound healing, organ failure and even death [5, 6]. Previous studies have documented that neuraxial anaesthesia attenuates the neuro-endocrine stress response after surgery [7–10]. The clinical relevance of this attenuation and any effects on PAMW after major surgery are not known. Hypothetically, attenuation of the neuro-endocrine stress response may affect PAMW and early mobilization postoperatively [11–13].

In a recent propensity matched analysis comparing SA to GA in more than 70,000 patients undergoing THA, Ferreira et al. found a significant reduced operative time in the SA group, as well as also found significantly less 30-day complications, readmissions, and reoperations. [14] Another recent propensity-matched cohort analyses demonstrated a small but significant increase in incidence of major complications, pneumonia and mortality with GA as opposed to SA [15]. SA was also associated with a significant decrease in postoperative surgical site infections after THA in a recent meta-analysis [16]. It is clear that further investigations are necessary to determine the role of SA in the perioperative management and outcomes of patients undergoing THA.

In this study, it was hypothesized that SA attenuates the neuro-endocrine stress response and the magnitude of PAMW to a greater extent than GA. This exploratory prospective randomized controlled trial was performed in a well-defined cohort of patients undergoing bilateral THA under either SA or GA.

## Methods

### Study population

This randomized controlled trial was approved by the Ethical Committee of Ziekenhuis Oost-Limburg, Genk (March 22, 2018-18/0010U-B371201835378) and was registered at clinicaltrials.gov (NCT03600454- July 26, 2018). Initially the hypothesis was planned to be tested in two independent, but complementary, studies in patients having bilateral THA (Arm 1;SA), and in patients having major abdominal surgery (Arm 2; epidural anaesthesia).

Due to implementation of Enhanced Recovery After Surgery (ERAS) protocols in major abdominal surgery patients in our hospital after approval of this study epidural anaesthesia was abandoned, preventing recruitment of subjects in Arm 2. Consequently, the protocol was amended to eliminate Arm 2. All surgical procedures were performed by one surgeon using the anterior approach. Inclusion criteria were: (1) patients aged 18 years or older (2) bilateral THA. Exclusion criteria were: (1) inability to understand or give an informed consent; (2) urgent surgery; (3) contra-indications for SA; (4) allergy or contraindications to any products used in the protocol; (5) history of chronic opioid use; (6) preoperative use of corticosteroids [less than 3 months before surgery]; (7) history of muscle wasting disease [e.g., Steinert disease, amyotrophic lateral sclerosis; Duchenne dystrophy].

Patient characteristics, American Society of Anesthesiologists (ASA) classification, comorbidities (diabetes mellitus, hypertension, coronary heart disease, renal insufficiency, liver cirrhosis, cancer), duration of surgery, total amount of anaesthetics administered, postoperative Numeric Rating Scale (NRS) pain scores, and total hospital length of stay were recorded. Written informed consent was obtained from each patient before inclusion in the study.

### Primary and secondary outcomes

The primary outcome variable was the change in peripheral limb muscle strength at postoperative day 1, 7 and 28, relative to baseline preoperative muscle strength. The baseline preoperative muscle strength is the muscle strength measured before surgery. The secondary outcomes were: (1) change in MIPat postoperative day 1, 7 and 28, (2) general health status as assessed by EQ-5D-5L questionnaires at postoperative day 1, 7 and 28 and (3) levels of markers of the neuro-endocrine stress and inflammatory response.

To reach sufficient statistical power (1-beta 0.80, based on a two-sided t-test with alpha 0.05) 22 subjects per group should be included. This power calculation was based on the assumption that a 10% decrease in muscle strength is clinically relevant in patients undergoing GA and that this could be reduced to a 7% drop in muscle strength when using SA with a SD of 3.5% [1]. A relative reduction of muscle weakness of 30% or more was considered as clinically relevant.

### Study design

Patients were enrolled between September 2018 and November 2019. Randomization was performed using a computer-generated permuted block randomization sequence (variable block-size, 1:1 allocation) and occurred during the preoperative anaesthesia assessment. Patients were randomized in two groups: (1) SA combined with monitored anaesthesia care (MAC); and (2) GA.

### Anaesthetic protocol

SA was performed thirty minutes before the onset of surgery using a standardized technique, consisting of 10 mg of intrathecal isobaric bupivacaine 0.5% administered at level L4–L5. During spinal anaesthesia, MAC was established with the administration of intravenous (IV) midazolam 1–2 mg and S-ketamine 5 mg. MAC was defined as the administration of anesthetics with the goal of patient comfort, while preserving meaningful patient contact. During surgery, MAC was administered to patients with IV propofol through target-controlled infusion (TCI); a target effect site concentration (EC) was maintained at 1.0 µg/ml (Marsh model).

During the induction of GA, patients received IV fentanyl 2 µg/kg and IV propofol through TCI; a target EC was maintained at 3.0 µg/ml (Marsh model) as maintenance of anaesthesia. Neuromuscular blockade was obtained with IV rocuronium 0.6 mg/kg. At the end of surgery, the neuromuscular blockade was reversed with the administration of IV sugammadex 4 mg/kg.

Intraoperatively, all patients received IV paracetamol 15 mg/kg (with a maximum dose of 1000 mg) and IV ketorolac 0.5 mg/kg (with a maximum dose of 30 mg). In the PACU, pain was treated by titrating IV piritramide 0.02 mg/kg, until the patient reported a NRS of 4 or less as per institutional protocol. On the ward, patients received IV paracetamol 15 mg/kg (with a maximum dose of 1000 mg) every 6 h and IV ketorolac 0.5 mg/kg (with a maximum dose of 30 mg) every 8 h. Intramuscular (IM) piritramide 0.2 mg/kg (maximum dose of 15 mg) up to four times a day was administered parenterally for NRS scores higher than 4. After oral intake was resumed, the oral analgesia protocol consisted of paracetamol 1000 mg each 6 h; diclofenac 75 mg each 12 h; tramadol slow-release 100 mg each 12 h; and tramadol fast-release 50 mg up to 4 times a day, when NRS were higher than 4.

### Postoperative protocol

All subjects followed the institutional protocol for patients undergoing bilateral THA. They were all admitted to the ICU overnight for continuous haemodynamic observation. On postoperative day one they were allowed to walk under supervision of the physiotherapist. They were allowed to leave the hospital on postoperative day 2. An outpatient follow-up visit was planned 6 weeks after the surgery.

### Peripheral limb muscle strength

Limb muscle strength was defined as the handgrip strength in the dominant hand and was measured using a handgrip dynamometer (Jamar® Plus Digital;Cognatus Innovations,LLC) with the patient in a sitting position and the elbow of the dominant arm in 90 degrees flexion. For each assessment, the average of three measurements was calculated and used in the analyses. Preoperatively, these values were normalized for age, gender and height using validated reference values (normalized strength = measured mean strength/reference value), which are shown in Table 1 for both groups [17]. For the rest of the analyses, relative changes compared to these baseline values are used or shown in figures.

**Table 1:** Baseline demographic and perioperative characteristics of the patients

	General anesthesia group *n* = 23	Spinal anesthesia group *n* = 23	*p*-value
Age (years)	63	63	0.54
Gender (M/F)	13/10	6/17	0.02
BMI (mean)	27	26	0.02
ASA physical status I/II/III (*n*)	8/12/3	10/10/3	0.74
Diabetes, *n* (%)	2 (9)	4 (17)	0.34
Hypertension, *n* (%)	4 (17)	6 (26)	0.26
Coronary and/or chronic heart disease, *n* (%)	3 (13)	1 (4)	0.16
Renal insufficiency, *n* (%)	0 (0)	1 (4)	0.22
Liver cirrhosis, *n* (%)	0 (0)	0 (0)	/
Cancer, *n* (%)	0 (0)	1 (4)	0.22
Active smoker, *n* (%)	1 (4)	1 (4)	0.97
Asthma, *n* (%)	0 (0)	1 (4)	0.22
COPD, *n* (%)	0 (0)	0 (0)	/
Mean duration surgery ± SD (min)	99 ± 23	96 ± 36	0.39
NRS on postoperative D1 Median (IQR)	2 (1–3)	1 (0–3)	0.61
Hospital length of stay—days median (IQR)	3.5 (3–6)	3 (3–5)	0.41
Normative handgrip strength ± SD (% of normal)	83 ± 21	77 ± 19	0.28
Normative MIP value ± SD (%of normal)	54 ± 24	52 ± 28	0.56
EQ5D-5L index Mean (SD)	0.81 (0.09)	0.78 (0.08)	0.30

*BMI* body mass index, *ASA* American Society of Anesthesiologists, *COPD* chronic obstructive pulmonary disease, *SD* standard deviation, *IQR* interquartile range, *NRS* numeric rating scale, *MIP* maximum inspiratory pressure

### Respiratory muscle strength

Respiratory muscle strength testing was performed using spirometry (Powerbreathe K3; POWERbreathe International Ltd.; UK). The MIP is a frequently used measure of the strength of inspiratory muscles, primarily the diaphragm, and allows for the assessment of ventilatory failure, restrictive lung disease and respiratory muscle strength and has been validated as a marker of ICUAW [18–20]. MIP was measured with the patient in sitting position. For each assessment, the average of three measurements was calculated and used in the analyses. Preoperatively, these values were normalized for age, gender and height using validated reference values (normalized MIP = measured mean MIP/reference value), which are shown in Table 1 for both groups [21]. For the rest of the analyses, relative changes compared to these baseline values are used or shown in figures.

### General health status

General health status was assessed using the EuroQol EQ-5D-5L questionnaire (Additional file 1). The descriptive system comprises five dimensions: mobility, self-care, usual activities, pain/discomfort and anxiety/depression. Each dimension has 5 levels: no problems, slight problems, moderate problems, severe problems and extreme problems. Handgrip strength, MIP and general health status were measured at four time points: (1) during the preoperative anaesthesia assessment, which served as a baseline measurement; (2) on postoperative day one; (3) and during a home visit on postoperative day 7 and (4) on postoperative day 28.

### Biochemical data

Since the neuro-endocrine and inflammatory stress response is known to play an important role in the development of ICUAW, the levels of the markers of these pathways, including the documented mediators concerning muscle function, HPA-axis and IL-6 were evaluated as these are validated markers of the surgically induced neuro-endocrine stress response and inflammatory response [5].

Blood was collected right before and at the end of surgery and 24 h after surgery in K_2_-EDTA tubes (BD Vacutainer, Ref 367864; BD, UK). Samples were centrifuged for 10 min at 1500 RCF and plasma was frozen at − 80 °C within 1 h after collection and kept frozen until analyzed. Analyses for ACTH, cortisol and IL-6 were performed on cobas e801. Quality controls were performed according to the ISO 15189 procedures of the central laboratory.

### Statistical analyses

Data are presented as means and standard deviations. Statistical analyses were performed with JMP version 15.0 (SAS Institute Inc., Cary, NC). Parametric comparisons between groups were made with the student t-test for normally distributed data. Normality of the data was evaluated using the Anderson–Darling test. Two-sided *p*-values of 0.05 or lower were considered statistically significant.

## Results

### Study population and patient characteristics

**Fig. 1:**
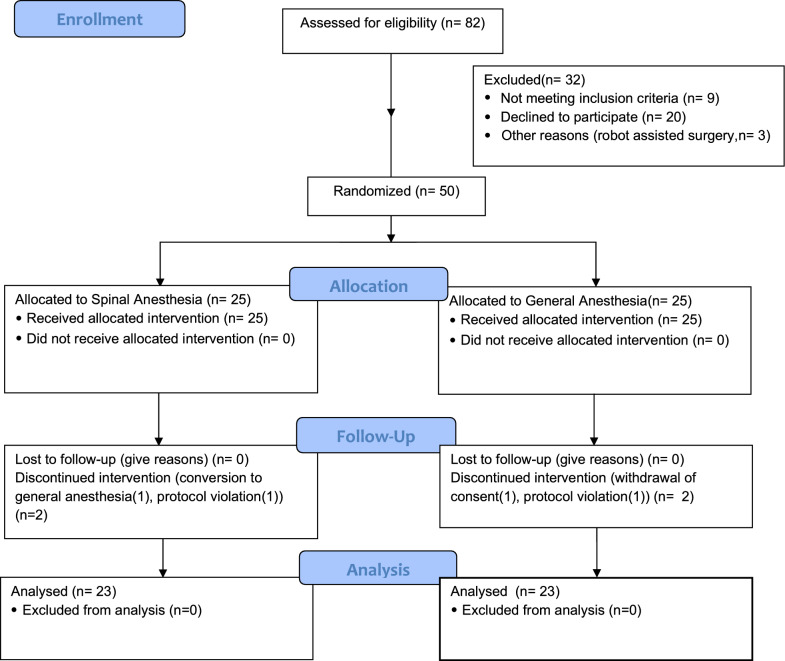
Consolidated standards of reporting trials diagram

A total of 50 patients were included and randomized. In the GA group, 1 patient withdrew consent and 1 patient received corticosteroids during surgery and was excluded due to protocol violation. In the SA group, 1 patient was converted to GA and 1 patient received peripheral blocks to SA, which was a protocol violation (Fig. 1). Therefore, the final analysis included a total of 46 patients, or 23 in each group. Patient demographics are shown in Table 1. No patients were lost to follow-up at day 7; 2 patients were lost to follow-up at day 28.

### Peripheral muscle function

Preoperative handgrip strength was comparable among both groups and was reduced to 82.72% (SD 20.78%) in the GA group and 76.63% (SD 18.79%) in the SA group (*p* = 0.28) as compared to their preoperatively baseline values (Table 1) [17].

**Fig. 2:**
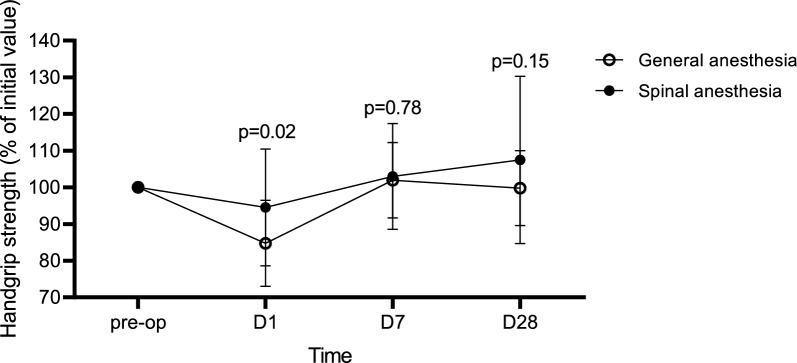
The evolution over time of changes in handgrip strength and the effect of type of anaesthesia. Mean and standard deviation of handgrip strength are shown on different time points as a percentage relative to baseline being the preoperative values and this for the different types of anaesthesia. *P*-values indicate the relative difference between the two types of anaesthesia on the different time points. *GA* general anaesthesia, *SA* spinal anesthesia, *MIP* maximum inspiratory pressure

Patients who received GA had a decrease in handgrip strength, as compared to their preoperative baseline values, by a mean 15.25% (SD 11.77%) on postoperative day 1, as opposed to a decrease of 5.49% (SD 15.96%) in the SA group (*p* = 0.02). Handgrip strength was restored to preoperative values by day 7 in both groups [+ 1.92% from baseline (SD 10.26%) in the GA group; + 2.98% from baseline (SD 14.40%) in the SA group (*p* = 0.78)]. At the 28-day follow-up, handgrip strength was comparable to preoperative values in both groups (0.25% (SD 10.22%) decrease in the GA group and a + 7.5% (SD 22.84%) from baseline in the SA group (*p* = 0.15). The changes of handgrip strength over time for each type of anaesthesia are shown in Fig. 2 and Table 2.

**Table 2:** Changes in handgrip strength and MIP and the effect of type of anaesthesia

	D1	D7	D28
**Handgrip strength**	**% of D0 baseline**	**% of D0 baseline**	**% of D0 baseline**
GA	84.75 (11.77)	101.92 (10.26)	99.75 (10.22)
SA	94.51 (15.96)	102.98 (14.40)	107.5 (22.84)
*p* value	0.02	0.78	0.15
**MIP**	**% of D0 baseline**	**% of D0 baseline**	**% of D0 baseline**
GA	87.76 (19.95)	114.28 (32.18)	127.77 (43.39)
SA	111.72 (48.3)	149.06 (47.88)	151.10 (60.05)
*p* value	0.04	0.006	0.16

Mean and standard deviation of handgrip strength and MIP are shown on different time points as a percentage relative to baseline being the preoperative values and this for the different types of anaesthesia. *P*-values indicate the relative difference between the two types of anaesthesia on the different time points. *GA* general anaesthesia, *SA* spinal anesthesia, *MIP* maximum inspiratory pressure

### Respiratory muscle function

The baseline MIP, adjusted for age and gender, was comparable between the two groups and was reduced as compared to their expected baseline values (Table 1) [21].

**Fig. 3:**
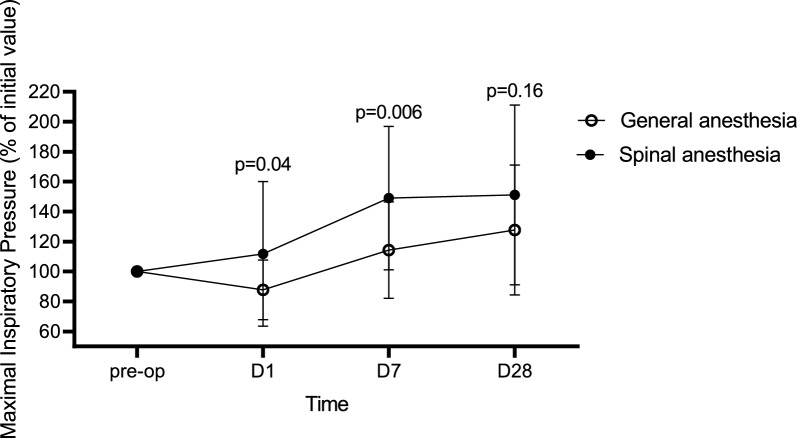
The evolution over time of changes in MIP and the effect of type of anaesthesia. Mean and standard deviation of MIP are shown on different time points as a percentage relative to baseline being the preoperative values and this for the different types of anaesthesia. P-values indicate the relative difference between the two types of anaesthesia on the different time points. *GA* general anaesthesia, *SA* spinal anesthesia, *MIP* maximum inspiratory pressure

Hip replacement surgery under GA was associated with a decrease in MIP, as compared to their baseline value, by a mean 12.24%, (SD 19.95%) on postoperative day 1, whereas MIP increased in the SA group by 11.72% (SD 48.30%) (*p* = 0.04). On day 7 MIP increased in both groups, as compared to the preoperative values. The increase in MIP was larger in patients receiving SA [mean increase 49.06% (SD 47.88%) in the SA group and 14.28% (SD 32.18%) in the GA group (*p* = 0.006)]. At the 28-day follow-up, MIP was increased in both groups as compared to preoperative values, however, without a statistically significant difference between groups, respectively, a 51.10% (SD 60.05%) increase in the SA group and a (27.77% (SD 43.39%) increase in the GA group (*p* = 0.16)**.** The evolution over time of postoperative MIP and the effect of anaesthesia are shown in Table 2 and Fig. 3. No correlation between the duration of surgery and MIP on postoperative day 1 was found.

### Health status

Median length of hospital stay was 3.5 days (IQR 3–6) in the GA group versus 3 days (IQR 3–5) in the SA group (*p* = 0.41).

**Fig. 4:**
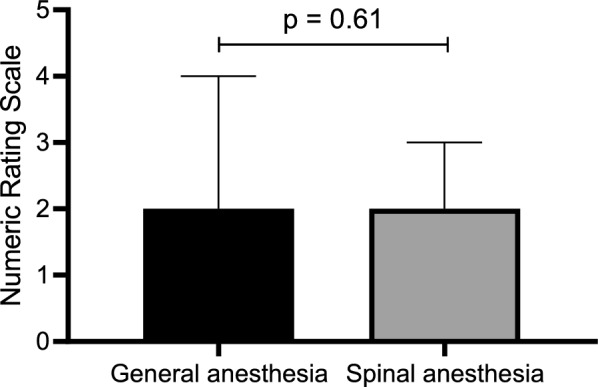
The Numeric pain scores on postoperative day 1 and the type of anaesthesia (*p* = 0.61)

The EQ5D-5L index was similar among both groups at baseline, with the mean EQ5D-5L index 0.81 (SD 0.09) in the GA group and 0.78 (SD 0.08) in the SA group (*p* = 0.30) (Table 1). On postoperative day 1, the EQ5D-5L index decreased by a mean 50.74% decrease in the GA group (SD 22.37%) and a mean 52.21% decrease in the SA group (SD 22.97%). This decrease was not associated with the type of anaesthesia (*p* = 0.82). Over the postoperative period, the EQ5D-5L index recovered, reaching similar preoperative values at follow-up day 28. Pain scores on postoperative day 1, during evaluation of the peripheral and lung muscle function, were comparable between the two groups (*p* = 0.61) (Fig. 4).

### Markers of neuro-endocrine stress response and inflammatory response (Table 3)

**Table 3:** Blood levels of markers of the neuro-endocrine and inflammatory stress response over time and the effect of type of anesthesia

		Pre-OP	End of surgery	Post-OP D1
ACTH (ng/l)	GA	32.9 (5.8)	72.8 (17.9)	27.5 (7.1)
	SA	34.7 (6.3)	27.1 (8.8)	18.9 (5.8)
	*p*-value	0.83	0.003	0.32
Cortisol (µg/dl)	GA	12.9 (0.9)	19.2 (1.3)	14.8 (1.8)
	SA	12.4 (1.1)	8.6 (1.1)	13.2 (1.7)
	*p*-value	0.70	< 0.0001	0.55
IL-6 (ng/l)	GA	6.24 (2.4))	43.08 (13)	369.8 (40.2)
	SA	3.45 (0.4)	15.81 (4.2)	345.1 (56.2)
	*p*-value	0.19	0.002	0.41

Mean values and standard deviations of ACTH, Cortisol and IL-6 are shown on different time points for the different types of anaesthesia. *p*-values indicate the relative difference between the two types of anaesthesia on the different time points

*ACTH* adrenocorticotropic hormone, *IL-6* interleukin-6, *GA* general anaesthesia, *SA* spinal anesthesia

**Fig. 5:**
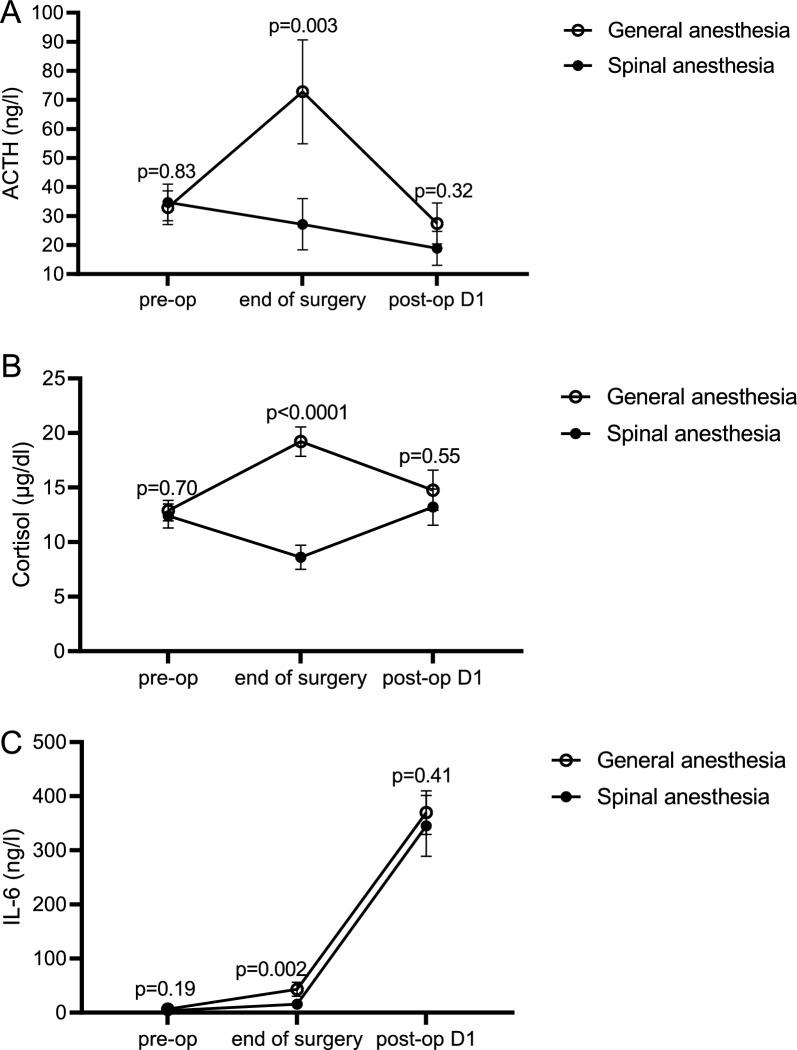
The evolution over time of changes of markers of the neuro-endocrine and inflammatory stress response and the effect of type of anesthesia. Mean values and standard deviations of ACTH, Cortisol and IL-6 are shown on different time points for the different types of anaesthesia. P-values indicate the relative difference between the two types of anaesthesia on the different time points. *ACTH* adrenocorticotropic hormone, *IL-6* interleukin-6

ACTH levels were elevated at the end of surgery in the GA group, but not in the SA group (*p* = 0.003) (Fig. 5). This elevated ACTH level was accompanied by elevation in cortisol level. The levels of ACTH and cortisol were not elevated in the SA group (*p* < 0.0001) (Fig. 5). Interestingly, on postoperative day 1, cortisol was similarly elevated in both groups (*p* = 0.54) with low ACTH levels.

IL-6 levels increased at the end of surgery twice as much in the GA group as compared to the SA group (*p* = 0.0029). The highest increase in IL-6 was seen on postoperative day 1, with no difference between both groups (*p* = 0.40) (Fig. 5).

## Discussion

This exploratory prospective randomized controlled trial demonstrated that patients who underwent bilateral hip replacement developed generalized muscle weakness postoperatively. Thereby note that a bilateral hip replacement is more invasive than a unilateral replacement. The degree of muscle weakness was greater in patients who received GA compared to patients who received SA. The SA patients also had a higher postoperative handgrip strength, MIP and lower levels of ACTH, cortisol, and IL-6 in the immediate postoperative period. The modulating effect on the neuro-endocrine pathway of neuraxial anaesthesia has been reported previously [22, 23]. Since it is known that several components of the neuro-endocrine stress response, such as cortisol, and the inflammatory response, for example IL-6, may induce muscle catabolism, the effect of the intervention on markers of these pathways was evaluated. SA yielded lower levels of ACTH, cortisol, and IL-6, but only in the immediate postoperative period on day 1. These data suggest that SA may modulate the immediate neuro-endocrine and inflammatory responses in patients having lower limb orthopaedic surgery. A recent systematic review, however, suggests that neuraxial anaesthesia does not influence IL-6 as compared to GA, but the interpretation of that review is limited by high heterogeneity [24]. In contrast, the current study has high internal validity (single center, only patients undergoing bilateral THA and one surgeon). Furthermore, measuring the levels of cytokines at only one time point might be insufficient to detect an effect. Therefore, further studies are needed to elucidate the effects of neuraxial anaesthesia on inflammatory pathways.

Handgrip dynamometry is an effective screening tool for global muscle weakness as a surrogate for the Medical Research Council (MRC) score and, it is associated with hospital mortality [25, 26]. In the current study, elective bilateral hip replacement under GA resulted in a 15% decrease in peripheral handgrip strength of the dominant arm. In the study by Lachmann et al., however, the PAMW persisted for more than one month, whereas in the current study the muscle strength was already restored to preoperative values after 7 days and this regardless of the type of anaesthesia [1]. However, the patients in the study of Lachmann et al. were generally older and underwent more invasive surgery [1]. In contrast, Petersson et al. did not find a decrease in peripheral muscle strength after elective cholecystectomy [27]. The discrepancy in these reports suggests that perioperative weakness may be lesser in young patients having less invasive surgery, but more severe and/or longer lasting in older patients having major surgery. This hypothesis, however, needs to be further substantiated. Nevertheless, patients who received SA in the current study had only a minor reduction in the muscle weakness (5% decrease in handgrip strength), or just a third of the reduction observed in the GA group (15% reduction). It is important to realize that the perioperative peripheral muscle weakness was only a transient effect, possibly influencing the postoperative period, but wasn’t sustained until one month after surgery. Therefore, the choice of anesthesia might influence the direct postoperative period, which is important in early recovery after surgery, but not in the long term.

Postoperative respiratory muscle function also appeared to recover faster in patients who had SA, as opposed to patients who received GA. In the GA group, there was a decrease in respiratory muscle strength on postoperative day 1, followed by a gradual recovery in MIP in the postoperative period. SA resulted in a better respiratory muscle strength in the direct postoperative period and this effect lasted during the following assessments on postoperative day 7 and day 28. It is well established that even a limited duration of mechanical ventilation may induce atrophy of the diaphragm, and that anaesthetics negatively affect the postoperative pulmonary function [28]. Regardless, the prolonged decrease in MIP that was documented on day 7 and day 28, could not cogently be explained. The reduced MIP at baseline in patients receiving bilateral hip replacement could be due to deconditioning.

The decrease in global health status in the immediate postoperative period, and its return to the pre-operative level after one month, was unaffected by the type of anaesthesia. The use of more sensitive questionnaires investigating fatigue and early recovery after surgery, such as QOR-15, in future larger studies could potentially clarify if this muscle strength preserving effect of SA translates in a better recovery after surgery.

Our study findings are in agreement with Lattermann et al. who reported that neuraxial anaesthesia decreases the catabolic stress response after surgery, and protein catabolism [7]. The reduction in muscle catabolism by modulating the stress response could be one of the factors that may be responsible for the lower effect seen on muscle strength in patients who received SA for bilateral THA. However, anaesthesia technique-related neuromuscular coupling/uncoupling and the central effects of anaesthesia on volitional testing cannot be excluded. Our data also confirm the findings of Lachmann et al. that surgery does indeed induce substantial muscle weakness [1]. To our knowledge, this study is the first to report a modulating effect of the type of anaesthesia on this PAMW. This may be of clinical importance, since reduction of PAMW may negatively affect early mobilization, rehabilitation, and ERAS protocols, therefore favoring SA in patients having bilateralTHA.

## Limitations

The effect of anaesthesia in bilateral total hip replacement surgery through the anterior approach was investigated in the current study. Therefore, these results should not be extrapolated to other surgical procedures. In addition, anterior approach to total hip arthroplasty is considered as “minimally invasive” and associated with a lower inflammatory response in comparison to traditional approaches [29]. Therefore, the difference between the GA and SA group could be different with traditional techniques for hip replacement. Moreover, dexamethasone or similar steroids were not administered in the perioperative phase although these medications reduce the surgical stress response and, therefore, could have an added value in the recovery of THA patients. Future studies need to evaluate the effect of glucocorticoids on postoperative muscle weakness and their impact on early recovery. Although the current study was adequately powered, a type 1 error is possible in smaller studies. Furthermore, since we investigated the change in muscle strength and not the muscle strength per se, we haven’t stratified for sex, BMI, or age since one wouldn’t expect a modulatory effect on muscle strength of these factors, however, one can’t exclude this possibility. Hence, larger confirmatory studies are needed to increase the external validity of the findings. Thirdly, while MIP and handgrip strength are commonly used as validated and objective measurements of muscle weakness, the effect of the intervention on other muscle related outcomes, such as a 6 min walking test, was not investigated. Moreover, the central effects of GA on the volitional nature of the MIP and PAMW measurements may have disfavored the GA group. Fourthly, our exploratory study was designed and powered to detect a difference in peripheral muscle weakness, a recently identified perioperative complication. However, larger, subsequent studies are necessary to study whether the observed effect on muscle weakness is associated with improved clinical outcomes, such as fatigue and fast tracking after surgery.

## Conclusion

In our study, in patients having bilateral THA, SA attenuated neuro-endocrine and inflammatory responses, and preserved the postoperative respiratory and peripheral muscle strength, compared to GA.

### Supplementary Information

**Additional file 1: **General health status was assessed using the EuroQol EQ-5D-5L questionnaire

## Data Availability

The datasets used and analyzed during the current study are available from the corresponding author on reasonable request.

## Abbreviations

ACTH: Adrenocorticotropic hormone
ASA: American Society of Anesthesiologists
BMI: Body mass index
COPD: Chronic obstructive pulmonary disease
EC: Effect site concentration
ERAS: Enhanced recovery after surgery
GA: General anaesthesia
ICUAW: Intensive care unit acquired muscle weakness
IL-6: Interleukin 6
IM: Intramuscular
IQR: Interquartile range
IV: Intravenous
MAC: Monitored anaesthesia care
MIP: Maximal inspiratory pressure
MRC: Medical Research Council
NRS: Numeric rating scale
PACU: Post anaesthesia care unit
PAMW: Perioperatively acquired muscle weakness
SA: Spinal anaesthesia
SD: Standard deviation
TCI: Target-controlled infusion
THA: Total hip arthroplasty
